# Optimizing Polychlorinated Biphenyl Degradation by Flavonoid-Induced Cells of the Rhizobacterium *Rhodococcus erythropolis* U23A

**DOI:** 10.1371/journal.pone.0126033

**Published:** 2015-05-13

**Authors:** Thi Thanh My Pham, Nancy Johanna Pino Rodriguez, Mohamed Hijri, Michel Sylvestre

**Affiliations:** 1 Institut National de la Recherche Scientifique, INRS-Institut Armand-Frappier, Laval, Québec, Canada; 2 Institut de Recherche en Biologie Végétale, Université de Montréal, Montréal, Québec, Canada; The University of Iowa, UNITED STATES

## Abstract

There is evidence that many plant secondary metabolites may act as signal molecules to trigger the bacterial ability to metabolize polychlorinated biphenyls (PCBs) during the rhizoremediation process. However, the bases for the PCB rhizoremediation process are still largely unknown. The rhizobacterium *Rhodococcus erythropolis* U23A is unable to use flavanone as a growth substrate. However, on the basis of an assay that monitors the amount of 4-chlorobenzoate produced from 4-chlorobiphenyl by cells grown co-metabolically on flavanone plus sodium acetate, this flavonoid was previously found to be a potential inducer of the U23A biphenyl catabolic pathway. In this work, and using the same assay, we identified ten other flavonoids that did not support growth, but that acted as inducers of the U23A biphenyl pathway, and we confirmed flavonoid induction of the biphenyl catabolic pathway using quantitative real-time polymerase chain reaction (RT-qPCR) on the *bphA *gene. We also examined the effect of the growth co-substrate on flavonoid induction. Sodium acetate was replaced by glucose, mannose, sucrose, or mannitol, which are sugars found in plant root exudates. The data showed that the level of induction of strain U23A biphenyl-degrading enzymes was significantly influenced by the nature and concentration of the flavonoid in the growth medium, as well as by the substrate used for growth. Sucrose allowed for an optimal induction response for most flavonoids. Some flavonoids, such as flavone and isoflavone, were better inducers of the biphenyl catabolic enzymes than biphenyl itself. We also found that all flavonoids tested in this work were metabolized by strain U23A during co-metabolic growth, but that the metabolite profiles, as well as the level of efficiency of degradation, differed for each flavonoid. To obtain insight into how flavonoids interact with strain U23A to promote polychlorinated biphenyl (PCB) degradation, we determined the concentration of flavanone at which optimal PCB-degrading performance of strain U23A was achieved. We showed that it corresponded to the concentration required to fully induce the biphenyl catabolic pathway of the strain. Together, our data demonstrate that optimal PCB degradation during the rhizoremediation process will require the adjustment of several parameters, including the presence of the appropriate flavonoids at the proper concentrations and the presence of proper growth substrates that positively influence the ability of flavonoids to induce the pathway.

## Introduction

Many investigations [1–10] have shown that plant secondary metabolites (PSMs), including flavonoids, may act as signal molecules to trigger the polychlorinated biphenyl (PCB)-degrading abilities of soil bacteria (for a review, see Singer [6]). As a result, PCB degradation is more efficient in vegetated soils when compared to non-vegetated ones [3, 5, 10, 11]. It has been postulated that this difference would be attributed to the presence of PSMs in vegetated soils, as released by plant roots [6], and of phenol derivatives generated by root turn-over [3]. This hypothesis is corroborated by at least one investigation, where it was clearly shown that PCB degradation by a PCB-degrading rhizobacterium was more efficient in soil microcosms seeded with wild-type *Arabidopsis thaliana* releasing phenylpropanoids than with those seeded with a flavonoid-null mutant [5]. However, in that study, the phenylpropanoids that promoted PCB degradation were not identified, and the mechanisms by which PSMs interact with bacterial metabolism to influence their ability to degrade PCBs in soil are still largely unknown.

Because of their structural similarities with biphenyl and the fact that the biphenyl catabolic pathway can metabolize several flavonoids, it was postulated that this pathway may play a role in modulating the quantity of PSMs in the rhizosphere [12]. In addition, these signal molecules may have a major impact on the success of the PCB-rhizoremediation process [13]. However, the bases for this process are largely unknown.

In a previous report, we showed that the rhizobacterium *Rhodococcus erythropolis* U23A, which was isolated from PCB-contaminated vegetated soil, carries a biphenyl catabolic pathway closely related to that of *Rhodococcus* sp. P6, described earlier [9]. Biphenyl-induced U23A cells metabolized the same range of PCB congeners as strain P6. These cells metabolized the same seven congeners of a mix of eighteen. Interestingly, U23A cells grown on *A*. *thaliana* root exudates were able to metabolize three of these seven congeners, whereas cells grown on a minimal medium containing sodium acetate as a growth substrate did not metabolize any of the tested congeners [9]. Flavanone, which has been identified as a major component of *A*. *thaliana* exudates, induced the biphenyl catabolic pathway of strain U23A when used as a co-substrate with sodium acetate [9]. Strain U23A was able to metabolize flavanone, but it could not grow on it because 4-oxo-2-chromanecarboxylic acid generated by the biphenyl catabolic enzymes was not further metabolized by the strain and had accumulated in the medium.

From these results, it was postulated that in vegetated soil, the rhizobacterium U23A may grow on labile chemicals (such as the sugar release from conjugated flavonoids), whereas the phenylpropanoid moiety may promote PCB degradation. A successful PCB-rhizoremediation process would then depend on the presence of flavonoids that act as inducers of the biphenyl catabolic pathway of PCB-degrading rhizobacteria during growth on the co-substrate. However, in the previous report, a limited number of flavonoids and only one co-substrate were tested for their ability to support growth and/or induce the biphenyl pathway of strain U23A.

In this work, we have examined the ability of a range of flavonoids to support the growth of strain U23A and/or to induce its biphenyl catabolic pathway. The data have shown that none of the tested flavonoids were able to support growth, but that several of them induced the biphenyl catabolic pathway when they were used as co-substrates with another available carbon source. To expand our knowledge about their potential to promote PCB degradation, we have determined the effect of the growth substrates on their ability to induce the biphenyl catabolic pathway, and we have also estimated the flavonoid concentrations required to reach the optimal PCB-degrading performance of strain U23A.

## Materials and Methods

### Bacterial strains, culture media, and chemicals

The bacterial strain used in this study was the rhizobacterium *R*. *erythropolis* U23A (ATCC BAA-2259) described earlier [9]. The culture media used were Luria—Bertani (LB) [14], basal medium M9 [14], or minimal mineral medium no.30 (MM30) [15] amended with various sources of carbon depending on the experiment. Strain U23A was maintained as glycerol stock at-80°C and it was revived on LB agar. The bacterium was grown at 28°C. Isoflavone, 7-hydroxyflavone, 6-hydroxyflavone, 4’-hydroxyflavanone, 6-hydroxyflavanone, 7-hydroxyflavanone, 3,7-dihydroxyflavone, 2’,3’-dihydroxyflavone, 2’,4’-dihydroxyflavone, 6,7-dihydroxyflavone, and 7,8-dihydroxyflavone were obtained from Indofine Chemical Company (Hillsborough, NJ, USA); all other chemicals used in the study were from Sigma-Aldrich Co. (St Louis, MO, USA). All standard stock solutions were prepared in acetone at concentrations ranging from 10–200 mM. The experiments described below to assess the ability of flavonoids to support growth or to induce the biphenyl catabolic pathway were performed in 10 mL Teflon-lined screw-capped vials. The flavonoids or biphenyl solutions were added to the sterile vials and the acetone was evaporated under a laminar flow cabinet prior to medium addition. Therefore, no acetone was present in the various assay media.

### Assessment of strain U23A growth on flavonoids or sugars

The ability of each flavonoid listed in Table 1 to serve as a growth substrate for strain U23A was assessed in MM30. Cells were grown overnight in MM30 amended with 30 mM of sodium acetate as a growth substrate. They were harvested by centrifugation and then washed in 0.85% NaCl and suspended in MM30 to an OD_600nm_ of 2.0. A volume of 0.1 mL of this suspension was used to inoculate 2 mL of MM30 amended with either one of the flavonoids listed in Table 1 at a concentration of 1 mM or 3 mM. The cultures were grown by shaking (150 rpm) at 28°C and colony-forming units on LB agar were monitored every day for 10 days. A similar assay was used to determine the ability of strain U23A to grow on either one of the various sugars listed in Table 1. In this case, the medium was supplemented with 30 mM of the sugar.

**Table 1:** List of flavonoids and sugars tested for their ability to support growth and/or induce the biphenyl catabolic pathway of strain U23A.

Compounds	Name				
Flavonoids	Flavanone	Flavone	Isoflavone	3-Hydroxyflavone	7-Hydroxyflavone
	6-Hydroxyflavone	4’-Hydroxyflavanone	6-Hydroxyflavanone	7-Hydroxyflavanone	3,7-Dihydroxyflavone
	2’,3’-Dihydroxyflavone	2’,4’-Dihydroxyflavone	6,7-Dihydroxyflavone	7,8-Dihydroxyflavone	Catechin
	Chrysin	Fisetin	Apigenin	Naringenin	Kaempferol
	Morin	Myricetin	Quercetin		
Sugars	Arabinose	Maltose	Mannose	Cellobiose	Galactose
	Rhamnose	Sucrose	Glucose	Xylose	Mannitol

### Assessment of the ability of flavonoids to induce the biphenyl pathway

A previously described resting cell assay [9] using 4-chlorobiphenyl as a substrate was used to assess the ability of various flavonoids to induce the biphenyl catabolic pathway of *R*. *erythropolis* U23A. Chlorobiphenyl metabolism was assessed by monitoring 4-chlorobenzoate, which accumulates as an end metabolite of the U23A biphenyl catabolic pathway. Briefly, cells were grown overnight in MM30 amended with 30 mM of sodium acetate or of one of the sugars that supports growth (listed in Table 1), in addition to 0.001–6 mM of any one of the flavonoids listed in Table 1 or of biphenyl. Controls containing only the individual sugars or sodium acetate were run in parallel. Cells were harvested, suspended, and adjusted to an OD_600nm_ of 1.0 with M9 without a carbon source. They were then distributed by portions of 200 μL into 1.5 mL Eppendorf tubes. Five microliters of 4-chlorobiphenyl in acetone (final concentration of 1.25 mM) or 5 μL acetone (negative control) was added and the reaction vials were incubated for 120 min at 28°C. The suspensions were then acidified and extracted with ethyl acetate. The extracts were derivatized with N,O-*bis*(trimethylsilyl)trifluoroacetamide (BSTFA) (Supelco; Sigma-Aldrich Co.) for gas chromatography—mass spectrometry (GC-MS) analysis [9]. The assays were performed in duplicate for each experimental setting.

### qPCR to confirm isoflavone induces expression of the biphenyl dioxygenase gene of strain U23A

To confirm that the flavonoids induced the biphenyl catabolic pathway of strain U23A, we used quantitative real-time polymerase chain reaction (RT-qPCR) analysis to quantify the expression of *bphA* in cells grown in sodium acetate plus isoflavone. This gene encodes the large subunit of biphenyl 2,3-dioxygenase that initiates biphenyl degradation. A 200 μL aliquot of U23A culture grown overnight in MM30 containing 30 mM of sodium acetate was used to inoculate 40 mL of MM30 amended with 30 mM of sodium acetate (as control) or 30 mM of sodium acetate plus 0.1 mM of biphenyl or isoflavone. Cells were grown by shaking at 100 rpm and at 28°C in a 200 mL Erlenmeyer flask; they were then harvested via mid-log phase (OD_600nm_ = 1.0) for RNA isolation. Total RNA was isolated by combining a three-step hot phenol protocol with the Qiagen RNeasy kit, as described by Atshan, Shamsudin [16]. Residual DNA digestion was performed on a silica gel column using RNase-free DNase. RNA was eluted in 20 μL of RNase-free water and the preparations were stored at-20°C. The quality and concentration of RNA preparations were determined using a Nano drop 2000 spectrophotometer. Reverse-transcription was performed using the Invitrogen SuperScript III Reverse Transcriptase. Then, quantitative polymerase chain reactions (qPCRs) were performed using the ViiA 7 Real-Time polymerase chain reaction (PCR) system (Life Technologies, Burlington, ON). The primers and TaqMan probes used for *bphA* and *gyrB* (housekeeping gene) are described in Table 2. Both TaqMan probes were labeled with FAM at 5’ end and nonflorescent HBQ1 at 3’ end (Alpha DNA, Montreal, QC). qPCR reactions were performed in volumes of 10 μL and they contained 6.25 ng of cDNA, 1.6 mM of each primer, 0.4 mM of TaqMan probe, 5 μL of iTaq Universal Probes Supermix (Bio-Rad, Mississauga, ON), and DEPC-treated water. PCR conditions were as follows: 10 min at 95°C, followed by 40 cycles of 15 sec at 95°C, and 1 min at 60°C. The controls that were run without DNA indicated that no ingredient contributed to the signal; moreover, the controls that were run with the isolated RNA indicated that contaminating DNA contributed to less than 1% of the fluorescence. All reactions were run in triplicate and three biological replicates were performed for each treatment. Data were processed according to Pfaffl [17] using cDNA isolated from U23A grown on sodium acetate only to prepare the calibration curve.

**Table 2:** Primers and probes used for qPCR.

Gene	Primer and probe	
*bphA*	Forward	CATGAGATCGAGGTGTGGT
	Reverse	GATCGTCTGACGCCTGTA
	Probe	ATGCTCCAGCCGAGATCAAGGAGG
*gyrB*	Forward	GTGGTACCCACGAAGAGG
	Reverse	TTGTCCTTGAGCAGCTTCT
	Probe	AACGGTCAACAAGTACGCGCTCGA

### Assay to assess flavanone’s ability to promote PCB degradation

A protocol using a mixture of 18 PCB congeners was used to assess the ability of flavanone, at various concentrations, to promote PCB degradation by strain U23A. The protocol was the same as the one described previously to evaluate the PCB-degrading performance of biphenyl-induced *R*. *erythropolis* U23A [9]. However, in this work, sucrose (instead of sodium acetate) was used as the co-substrate with flavanone. Briefly, cells were grown overnight at 28°C on MM30 amended with either 3.4 mM of biphenyl, 30 mM of sucrose, 30 mM of sucrose plus 3.4 mM of biphenyl, or 30 mM of sucrose plus 1, 0.1, or 0.001 mM of flavanone. The concentrations for sucrose and biphenyl were the same as those used in a previous study [9], whereas flavanone concentrations were chosen on the basis of the data obtained from the 4-chlorobiphenyl degradation assay described above. Overnight-grown cultures were filtered through packed glass wool to remove substrate crystals. Cells were harvested and suspended in MM30 to an OD_600nm_ of 1.0 and then distributed in 2 mL Teflon-lined screw-cap tubes in portions of 200 μL containing 1 μL of the mixture of the eighteen PCB congeners prepared in ethanol, as described previously [9]. Assays were performed in duplicate for each experimental setting. Tubes were incubated overnight at 28°C and shaken at 100 rpm. The cultures were then extracted twice with hexane [9]. The extracts were analyzed by gas chromatography equipped with an electron capture detector to quantify PCB depletion [18]. The percent depletion for each congener was calculated relative to a set of control tubes without cells extracted immediately following PCB addition and using an internal standard, as described previously [9].

### Flavonoid metabolism during co-metabolic growth

U23A cells were grown overnight in MM30, amended with sodium acetate, and they were harvested, washed, and suspended in MM30 to an OD_600mn_ of 1.0. This suspension (0.1 mL) was used to inoculate 2 mL of fresh MM30 containing 30 mM of sucrose plus 0.2 mM of either flavone, isoflavone, flavanone, 3-hydroxyflavone, 6-hydroxyflavone, 7-hydroxyflavone, 7-hydroxyflavanone, 3,7-dihydroxyflavone, chrysin, or fisetin. The cultures were incubated for 18 h at 28°C with shaking (150 rpm), and they were then centrifuged. The supernatant was extracted with ethyl acetate at neutral and acidic pH using a protocol described previously [12]. The extracts were treated with BSTFA for the GC-MS analyses [12]. The metabolites were tentatively identified on the basis of their GC-MS spectra by comparison with the previously described spectral features of metabolites produced from flavanone by *R*. *erythropolis* U23A [9] or from flavone, flavanone, or isoflavone by *Pandoraea pnomenusa* B356 and *Burkholderia xenovorans* LB400 [12].

## Results and Discussion

### Ability of flavonoids to induce U23A biphenyl pathway

**Fig 1:**
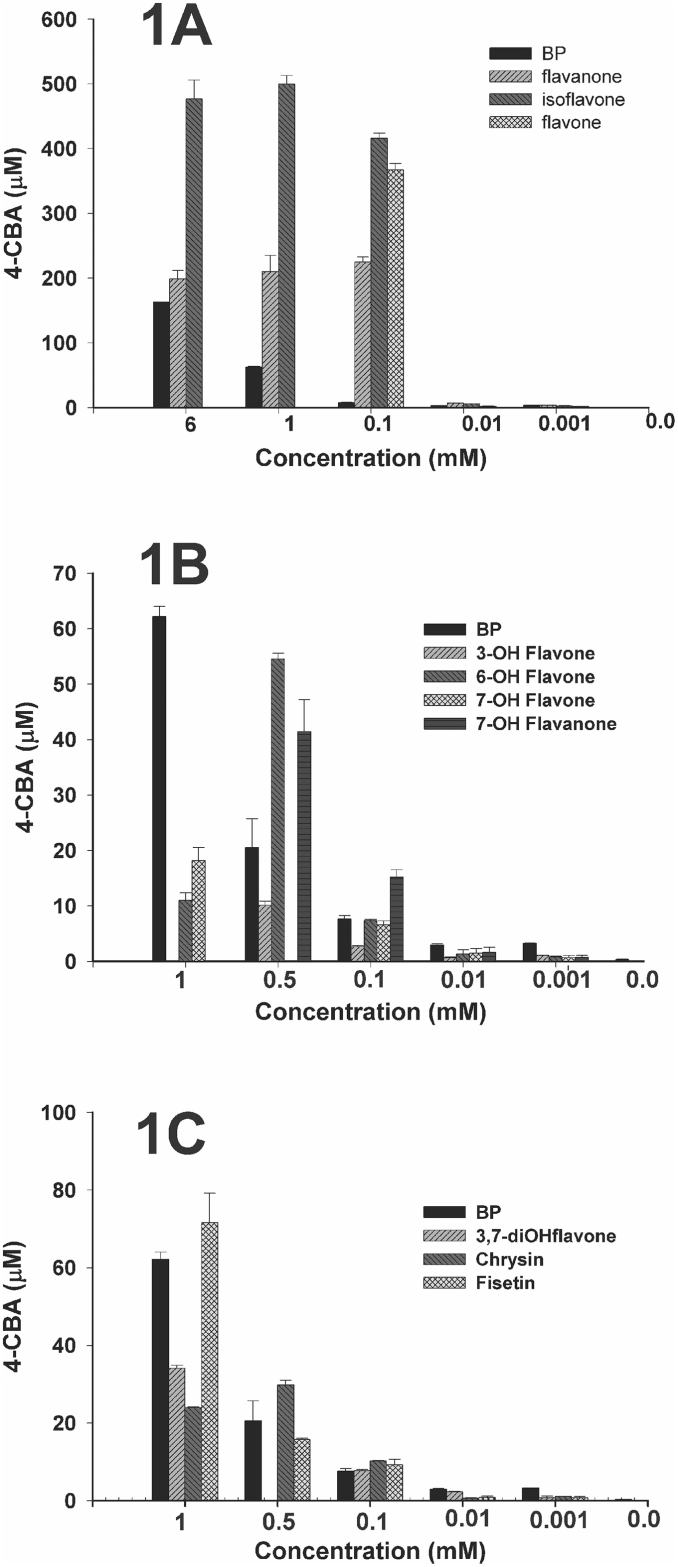
Amount (μM) of 4-chlorobenzoic acid produced when standardized resting cell suspensions of strain U23A were incubated with 1.25 mM 4-chlorobiphenyl for 2 h. **(A)** Cells were previously grown in MM30 amended with 30 mM sodium acetate plus the indicated concentrations of biphenyl or each of the simple flavonoids (cells did not grow in culture containing 1 and 6 mM flavone). **(B)** Cells were previously grown in MM30 amended with 30 mM sodium acetate plus the indicated concentrations of biphenyl or each of the monohydroxy-flavone or-flavanone (0.5 mM 7-hydroxyflavone and 1 mM 3-hydroxyflavone were not tested). **(C)** Cells were previously grown in MM30 amended with 30 mM sodium acetate plus the indicated concentrations of biphenyl or each of the dihydroxyflavone (0.5 mM 3,7-dihydroxyflavone was not tested). Bars represent mean (n = 3) and SD are shown.

None of the flavonoids listed in Table 1 were able to support the growth of strain U23A. In a previous work [9] that used an assay monitoring the amount of 4-chlorobenzoate produced from 4-chlorobiphenyl, we reported that U23A cells grown co-metabolically on sodium acetate plus flavanone produced more 4-chlorobenzoate than those grown on sodium acetate alone. This suggested that flavanone induced the biphenyl catabolic pathway [9]. In the present work, and using the same assay, we identified ten potential inducers of the strain U23A biphenyl catabolic pathway among the flavonoids listed in Table 1. Fig 1A, 1B and 1C show the amount of 4-chlorobenzoate produced from 4-chlorobiphenyl for various concentrations of each of these ten flavonoids. A one-way ANOVA was run to analyze the data. For all flavonoids, at 0.1 mM, the amount of 4-chlorobenzoate produced was significantly higher (p<0.01) than for the control grown on sodium acetate alone. However, at 0.001 mM, flavone, flavanone, and isoflavone were the only ones for which 4-chlorobenzoate production was significantly higher than the control without the inducer. It is noteworthy that at 0.1 mM, the induction caused by these three flavonoids was significantly higher (p<0.01) than for biphenyl.

To confirm that U23A *bph* genes were induced, we used RT-qPCR analyses to determine the level of *bphA* expression in U23A cells grown on 0.1 mM of isoflavone plus 30 mM of sodium acetate. The data showed that *bphA* was upregulated by a factor of 13.4 ± 1 (p<0.001) relative to a control without the inducer. It is noteworthy that, as a comparison, *bphA* expression for cells grown on 0.1 mM of biphenyl plus sodium acetate was upregulated by a factor of 1.3 ± 0.6, only relative to the uninduced control. This confirms the ability of isoflavone to induce the biphenyl dioxygenase of strain U23A and provides evidence that in the 4-chlorobenzoate assay described above, 4-chlorobiphenyl was transformed by the biphenyl catabolic pathway enzymes of strain U23A.

It is also worth mentioning that the amount of 4-chlorobenzoate produced from 4-chlorobiphenyl was previously reported to be much higher (reaching concentrations above 800 μM) when cells were pre-grown on biphenyl alone than when they were pre-grown co-metabolically on biphenyl plus sodium acetate (not shown) [9]. Although we cannot exclude that sodium acetate may exert catabolite repression similar to that observed for the biphenyl catabolic pathway of *Acidovorax* sp. KKS102[19], many other factors may have been responsible for this observation. 4-Chlorobenzoate production may be influenced by post-transcriptional regulation factors, as well as by the physiological state of cells. Nevertheless, when cells were grown on sodium acetate plus 0.001–6 mM of biphenyl, the amount of 4-chlorobenzoate produced from 4-chlorobiphenyl correlated with the biphenyl concentrations (R^2^ = 0.997). This confirmed that the level of enzyme expression depended on the amount of biphenyl available to interact with the transcriptional regulatory system.

Unlike cells grown co-metabolically on biphenyl plus sodium acetate, the amount of 4-chlorobenzoate produced from 4-chlorobiphenyl by cells co-metabolically grown on flavone, isoflavone, or flavanone plus sodium acetate plateaued or decreased in concentrations above 0.1 mM (Fig 1A). Our data did not enable us to identify the mechanisms responsible for this apparent inhibition. Nevertheless, our results show that the level of expression or activity of the biphenyl catabolic enzymes may vary considerably depending on the nature, as well as the concentration, of the flavonoids on which strain U23A is co-metabolically grown. Therefore, we may conclude that the optimal degradation of chlorobiphenyls may be affected by the choice and concentrations of the flavonoids used for co-metabolic growth.

### Influence of growth substrate on biphenyl pathway induction

In the set of experiments described above, sodium acetate was a convenient growth substrate that was used to examine the ability of flavonoids to induce the biphenyl catabolic pathway of strain U23A. However, although short-chain aliphatic acids are expected to be present in the rhizosphere [20], other potential growth substrates (including many sugars) may also be released in plant exudates or they may be produced from root debris degradation [21]. Therefore, sugars may also be appropriate growth co-substrates that can be used along with potential flavonoid inducers. Furthermore, previous investigations have shown that carbon sources used during the co-metabolic degradation of PCB significantly influence the expression of the bacterial biphenyl catabolic pathway [22]. Furthermore, each PCB-degrading bacterium may respond differently to the environmental factors that influence the peripheral pathways involved in PCB degradation [23]. We thus tested the effect of using growth co-substrates other than sodium acetate on the ability of flavonoids to induce the biphenyl catabolic pathway of strain U23A.

**Fig 2:**
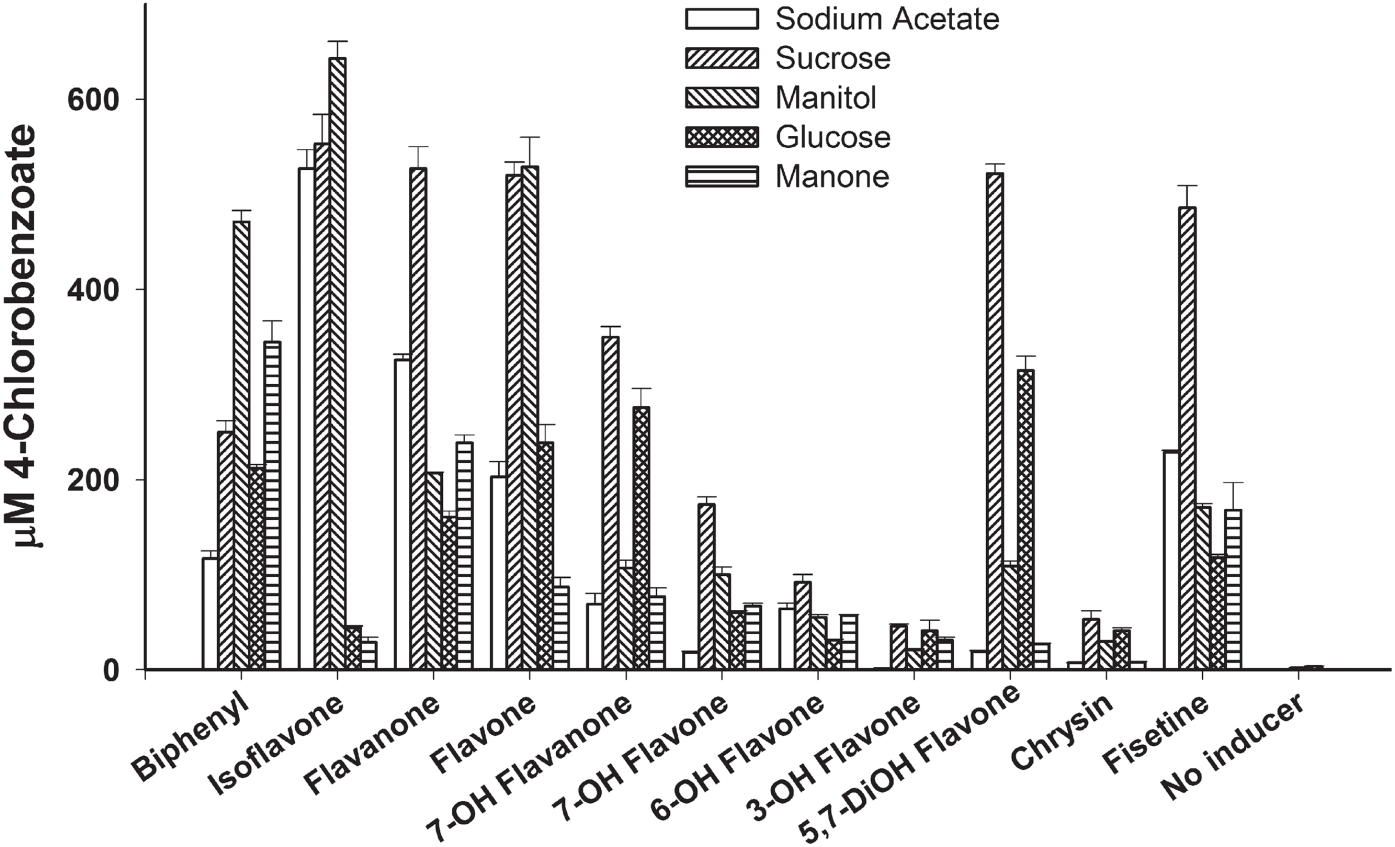
Amount (μM) of 4-chlorobenzoic acid produced when standardized resting cell suspensions of strain U23A were incubated with 1.25 mM 4-chlorobiphenyl for 2 h. Cells were previously grown in MM30 amended with 30 mM sodium acetate alone or 30 mM of the indicated sugars plus 3 mM biphenyl or each of the indicated flavonoids at the concentration that gave optimal induction response in the experimental setting of Fig 1 (for example, chrysin and fisetin were added at 1 mM). Bars represent mean (n = 2) and SD are shown.

The amount and nature of the sugars released in the exudates are subject to many factors, including the soil structure, microbial population of the rhizosphere, nutrients, plant developmental stage, and plant species [24]. Furthermore, there are scarce reports describing the precise nature of the sugars released in plant exudates. In this investigation, we selected those sugars (listed in Table 1) that were reported as being present in the root exudates of at least one plant species among rice, maize, and *Arabidopsis* [5, 24–26]. As a first step, we determined the carbohydrate utilization profile of strain U23A among the sugars listed in Table 1. Sucrose, mannitol, glucose, and mannose were the only ones that supported growth. Consequently, we used these four sugars to assess the ability of the ten flavonoids that were highlighted in the aforementioned experiments to induce the biphenyl catabolic pathway of the strain. The data are presented in Fig 2.

For all flavonoids, the amount of 4-chlorobenzoate produced from 4-chlorobiphenyl was significantly higher (one-way ANOVA; p<0.05) than for the controls without flavonoids. Furthermore, according to Tukey’s test for each individual flavonoid, the induction of the biphenyl catabolic pathway varied significantly depending on the carbon source used for growth (data not shown). As an example, isoflavone was a strong inducer when cells were co-metabolically grown on sodium acetate, sucrose, or mannitol, but the amount of 4-chlorobenzoate produced by cells grown on glucose or mannose plus isoflavone was significantly lower (p<0.01). We did not further explore the factors that may have influenced the ability of the various flavonoids to induce the catabolic pathway when used as co-substrates with each of the tested sugars. Nevertheless, sucrose was the one sugar on which cells produced a high level of 4-chlorobenzoate for most flavonoids.

### Flavonoid metabolism during co-metabolic growth on sucrose

Flavonoid metabolism may affect their ability to promote PCB degradation in two ways. If the flavonoid is metabolized rapidly by the strain, then at low concentrations, premature depletion during growth may hinder its ability to induce the biphenyl catabolic pathway. On the other hand, we cannot exclude the possibility that some of the flavonoid metabolites may repress production or inhibit the biphenyl catabolic enzymes of strain U23A. Therefore, since the outcome of the rhizoremediation process may be affected by the ability of the PCB-degrading strain to metabolize the flavonoids, which serve as inducers of the biphenyl catabolic pathway, we have examined the metabolism of selected flavonoids during the co-metabolic growth of strain U23A. In this study, sucrose was chosen as a growth substrate since the previously reported data indicated that sucrose-grown cells produced a high level of 4-chlorobenzoate for most flavonoids. Overnight cultures of co-metabolically grown cells were extracted with ethyl acetate at neutral and acidic pH, and the metabolites were analyzed by GC-MS.

When the cells were grown overnight with 0.2 mM of flavanone as the co-substrate, 0.15 mM of the substrate was depleted after 18 h and most of it was transformed to a metabolite that exhibited spectral features identical to those reported for 4-oxo-2-chromanecarboxylic acid [12] (Table 3). This is consistent with previous results [9] that showed that the U23A strain converted flavanone to 4-oxo-2-chromanecarboxylic acid, which was produced as a dead-end metabolite.

**Table 3:** Metabolites produced by strain U23A during co-metabolic growth on simple flavonoids.

Flavonoid	Metabolites^a^ ^,^ ^b^	Spectral features (TMS derivatives)			Comment
		M^+^	M-15	selected ions	
Flavanone	4-oxo-2-chromanecarboxylic acid	264	249	219, 205, 177, 147, 131	**Major metabolite**
	3’ or 2’-monohydroxyflavanone	310	295	265, 239	Trace
Flavone	4-oxo-2-chromenecarboxylic acid	262	247	217, 203, 195, 177, 145, 133	**Major metabolite**
	2’,3’- or 3’,4’-dihydroxyflavone	398	383	353, 310, 220	Trace
Isoflavone	4-oxo-3-chromenecarboxylic acid	262	247	217, 203, 173, 145	**Major metabolite**
	2’,3’- or 3’,4’-dihydroxyisoflavone	398	383	353, 310, 219	Trace

^a^ Strain U23A was grown on sodium acetate plus anyone of the indicated flavonoids and the metabolites were extracted and analysed by GC-MS as described in the Materials and Methods section.

^b^ For position numbering of metabolites refer to Fig 1.

Consistent with the observation that isoflavone and flavone were also strong inducers of the biphenyl catabolic pathway, when they were used as co-substrates with sucrose, they both transformed very efficiently. For each of them, the major metabolite exhibited spectral features that were quite similar to those of 4-oxo-2-chromanecarboxylic acid, with the exception of the molecular ion, which was at *m/z* 262 instead of 264. They were thus respectively identified as 4-oxo-3-chromenecarboxylic and 4-oxo-2-chromenecarboxylic acids (Table 3). In addition, both isoflavone and flavone produced metabolites that exhibited spectral features that would be expected for hydroxylated derivatives, and which were similar to the metabolites that were previously observed when flavanone was the co-substrate [9]. According to previous reports performed with other strains [12, 27, 28], the flavonoid substrates were presumably hydroxylated onto ring B (Table 3). However, on the basis their GC-MS peak area, these metabolites represented less than 1% of the metabolites detected in the growth medium. Together, the data showed that flavone and isoflavone were metabolized efficiently by all four enzymes of the biphenyl catabolic pathway to ultimately generate the oxo-chrome(a)ne carboxylic acid, which was not further metabolized by the cells.

**Fig 3:**
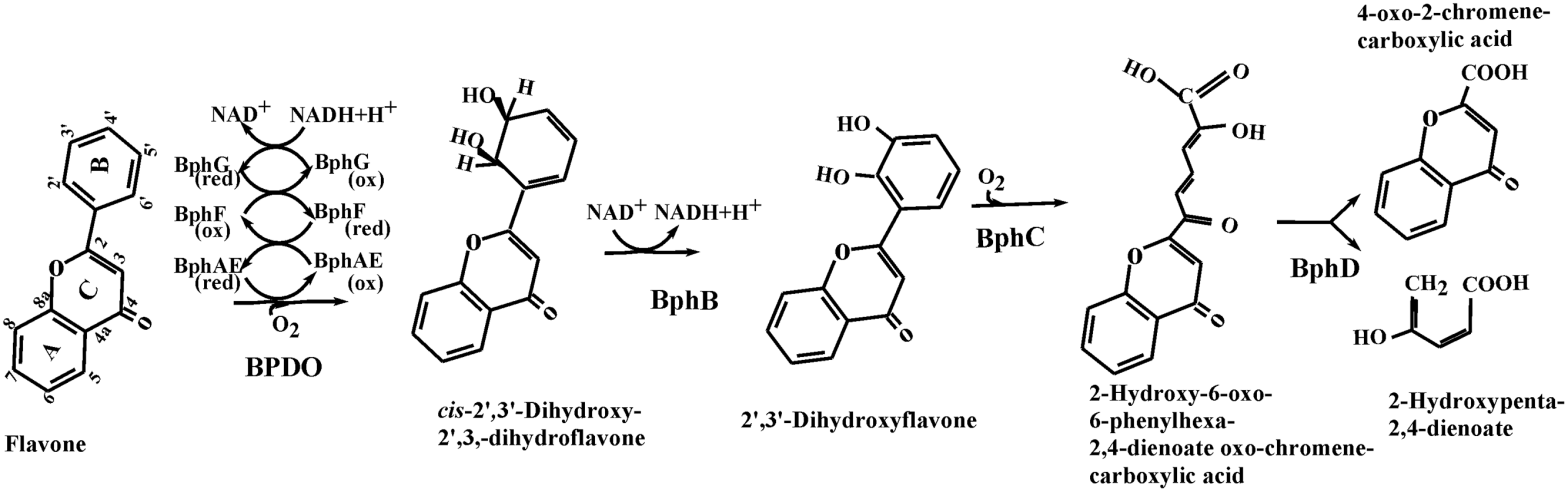
Proposed pathway for the production of 4-oxo-2-chromene carboxylic acid from flavone by strain U23A biphenyl catabolic enzymes. Data is based on metabolites detected in 18 h-old culture of strain U23A co-metabolically grown on sucrose plus flavone plus metabolites detected in resting cell suspensions of biphenyl-induced U23A cells incubated for variable periods of time with flavone.

As a representative illustration of the pathway, Fig 3 shows the enzymatic steps that generated 4-oxo-2-chromenecarboxylic acid from flavone. Although not all pathway intermediates were detected in the 18 h-old cultures of strain U23A grown on sucrose plus flavone, all of them were detected in biphenyl-induced U23A resting cell suspensions that were incubated for a short period of time with flavone (data not shown). The scarcity of metabolites, other than oxo-chromenecarboxylic acid, in the 18 h-old cultures of co-metabolically grown cells confirms that the metabolism was very efficient for these flavonoids.

Among the hydroxylated flavonoids, 3-hydroxyflavone was the least efficient inducer of the biphenyl catabolic pathway enzymes (Fig 1B). Consistent with this result, strain U23A cells weakly transformed 3-hydroxyflavone during co-metabolic growth on sucrose. Less than 1% of the substrate was converted after 18 h of incubation. Most of the substrate was converted to a metabolite that exhibited mass spectral features of a monohydroxylated 3-hydroxyflavone (Table 4). Only traces of a hydroxychromanecarboxylic acid (presumably 3-hydroxy-4-oxo-2-chromenecarboxylic acid) were also detected. On the other hand, 6-hydroxyflavone, a potent inducer of the U23A biphenyl pathway (Fig 1B), was metabolized significantly more efficiently than 3-hydroxyflavone during growth on sucrose. Based on the substrate peak area, approximately 15% of 6-hydroxyflavone was metabolized in the 18 h-old culture. Two metabolites were produced in approximately equal amounts: one exhibited the mass spectral features of a chromanecarboxylic acid (most likely 6-hydroxy-4-oxo-2-chromenecarboxylic acid), and the other exhibited features of a monohydroxylated derivative (Table 4). The monohydroxylated derivative was most likely generated from the dihydro-dihydroxyl metabolite of the dioxygenation reaction following water removal. However, we cannot exclude a catalytic monooxygenation reaction similar to the one observed previously when isoflavan-4-ol was the substrate for *Pseudomonas pseudoalcaligenes* KF707 biphenyl dioxygenase [29].

**Table 4:** Metabolites produced by strain U23A during co-metabolic growth on mono-hydroxyflavones.

Flavonoid^b^	Metabolites^a^ ^,^ ^b^	Spectral features (TMS derivatives)			Comment
		M^+^	M-15	selected ions	
**3-Hydroxyflavone**	3-hydroxy-4-oxo-2-chromenecarboxylic acid	350	335	262, 219, 205, 147, 131	Trace
	3’- or 4’-monohydroxylated 3-hydoxyflavone	398	383	355, 310, 219	**Major metabolite**
**6-Hydroxyflavone**	6-hydroxy-4-oxo-2-chromenecarboxylic acid	350	335	262, 219, 147, 131	50% of metabolites
	monohydroxylated 6-hydoxyflavone	398	383	355, 310, 219	50% of metabolites
**7-hydroxyflavone**	7-hydroxy-4-oxo-2-chromenecarboxylic acid	350	335	262, 219, 203, 147, 131	Trace
	3’- or 4’-monohydroxylated 6-hydoxyflavone	398	383	355, 310, 219	Trace

^a^ Strain U23A was grown on sodium acetate plus anyone of the indicated flavonoids and the metabolites were extracted and analysed by GC-MS as described in the Materials and Methods section.

^b^ For position numbering of flavonoids and metabolites refer to Fig 1.

Although 7-hydroxyflavone was also a potent inducer of the biphenyl pathway (Fig 1B), it was poorly metabolized during the growth of U23A on sucrose combined with this flavonoid. Only traces of a metabolite exhibiting the mass spectral features of a chromanecarboxylic acid, as well as of another metabolite exhibiting the features of a monohydroxylated metabolite, were detected after 18 h of growth (Table 4).

On the other hand, among the tested monohydroxylated flavonoids, 7-hydroxyflavanone was the one that strain U23A metabolized most efficiently. Approximately 25% of the added substrate was converted. The major metabolite, representing 80% of the total metabolites, was identified as a monohydroxylated derivative (Table 5). Traces of a dihydroxylated derivative, which may have been 2’,3’,7- or 3’,4’,7-trihydroxyflavanone, were also detected (Table 5). Trihydroxyflavanone was further metabolized since approximately 20% of the total metabolites consisted of a hydroxylated 4-oxo-2-chromancarboxylic acid, which was most likely 7-hydroxy-4-oxo-2-chromanecarboxylic acid (Table 5).

**Table 5:** Metabolites produced by strain U23A during co-metabolic growth on 7-hydroxyflavanone.

Flavonoid^b^	Metabolites^a^ ^,^ ^b^	Spectral features (TMS derivatives)			Comments
		M^+^	M-15	selected ions	
7-Hydroxyflavanone	3’- or 4’-monohydroxy-7-hydroxyflavanone	400	385	369, 341, 307, 281, 177	**Major metabolite**
	2’,3’ or 3’-4’-dihydroxy 7-hydroxyflavanone	488	473	400, 385, 370, 312, 293, 264	Trace
	7-hydroxy-4-oxo-2-chromanecarboxylic acid	352	337	307, 293, 265, 235, 219, 147	20% of metabolites

^a^ Strain U23A was grown on sodium acetate plus anyone of the indicated flavonoids and the metabolites were extracted and analysed by GC-MS as described in the Materials and Methods section.

^b^ For position numbering of flavonoids and metabolites refer to Fig 1.

The observation that 7-hydroxyflavone and 7-hydroxyflavanone were metabolized very differently by strain U23A suggests that the presence of a double bond onto carbons 2 and 3 may strongly influence the interaction between the biphenyl dioxygenase of strain U23A and the flavonoids hydroxylated onto carbon 7. The inability of strain U23A to metabolize 7-hydroxyflavone suggested that the presence of a hydroxyl group onto carbon 7 may hinder catalytic oxidation of the flavone substrates via biphenyl dioxygenase or one of the subsequent enzymes in the pathway. This is consistent with a previous report showing that the biphenyl dioxygenase of the PCB-degrading *P*. *pseudoalcaligenes* KF707 was unable to oxidize 7-hydroxyflavone [30]. It would also explain why 3,7-dihydroxyflavone, which was a good inducer of the biphenyl catabolic pathway (Fig 1C), was not metabolized by the strain during co-metabolic growth. The substrate was not depleted and no metabolites were detected in the 18 h-old culture grown in the presence of this substrate (Table 6). Also consistent with this observation, chrysin (5,7-dihydroxyflavone) and fisetin (3,7,3’,4’-tetrahydroxyflavone) were both poorly metabolized by strain U23A; this was in spite of the fact that fisetin was a good inducer of the biphenyl catabolic pathway (Fig 1C). In the case of chrysin, only about 1% of the added substrate was converted to a metabolite that exhibited features of a dihydroxy-oxo-chromenecarboxylic acid, which may have been 5,7-dihydroxy-4-oxo-2-chromenecarboxylic acid (Table 6). The ethyl acetate extract also contained traces of a metabolite exhibiting the mass spectral features of a tetrahydroxylated flavones, which could have been 5,7,2’,3’- or 5,7,3’,4’-tetrahydroxyflavone. Fisetin generated only one metabolite, a monohydroxylated derivative that was detected in trace amounts in the 18 h-old culture, and which we presumed was hydroxylated on the B ring (Table 6). The inability of strain U23A to metabolize fisetin was confirmed by the fact that 99% of the substrate remained in the 18 h-old culture.

**Table 6:** Metabolites produced by strain U23A during co-metabolic growth on di- or tri-hydroxyflavones.

Flavonoid^b^	Metabolites^a^ ^,^ ^b^	Spectral features (TMS derivatives)			Comment
		M^+^	M-15	selected ions	
3,7-Dihydroxyflavone					**No metabolite**
Chrysin	5,7-dihydroxy-4-oxo-2-chromenecarboxylic acid	438	423	351, 321, 219, 147	**Major metabolite**
	2’,3’- or 3’,4’-dihydroxychrysin	574	559	486, 398, 383, 370, 326, 264, 219, 131	Trace
Fisetin	2’- or 3’-2’- or 3’-monohydroxyfisetin	662	647	574, 559, 486, 471, 446, 398, 222, 207	**Sole metabolite**

^a^ Strain U23A was grown on sodium acetate plus anyone of the indicated flavonoids and the metabolites were extracted and analysed by GC-MS as described in the Materials and Methods section.

^b^ For position numbering of flavonoids and metabolites refer to Fig 1.

### PCB-degrading performance of flavanone-induced U23A cells

In a previous study using a resting cell assay to determine the ability of strain U23A to metabolize a mixture of 18 PCB congeners, it was shown that cells grown on *Arabidopsis* root exudates were able to metabolize three congeners that were not metabolized by cells grown on sodium acetate alone [9]. However, the range of PCB congeners degraded by cells grown on root exudates was smaller than that for cells grown on biphenyl as a sole growth substrate. This suggested that full induction of the biphenyl catabolic pathway is required to reach the optimal PCB-degrading performance of cells, which was consistent with previous observations showing that the range of biphenyl analogs that the biphenyl dioxygenase can metabolize increases with enzyme concentration [31, 32].

In order to determine the flavonoid concentrations that would confer optimal PCB-degrading performance to the cell, we have examined the PCB-degrading ability of strain U23A cells grown co-metabolically on sucrose plus variable concentrations of flavanone. We chose sucrose as the growth substrate since this sugar was among the tested co-substrates; in addition, it was the one for which most of the tested flavonoids exerted their highest ability to induce the biphenyl catabolic pathway. We chose flavanone as a flavonoid co-substrate since it was one of the most effective inducers among the tested flavonoids.

As shown in Table 7, and consistent with previous reports [9], among the eighteen congeners tested, seven were metabolized by fully induced U23A cells (grown on biphenyl alone). Also consistent with the data presented above, when cells were grown on sucrose plus biphenyl, cells were not fully induced and the % depletion of each of these congeners (except for 2,4,3’- and 2,3’,4,4’-chlorobiphenyl) was significantly lower than that for fully induced cells (Table 7). At 0.001 mM, flavanone induced the biphenyl catabolic pathway at a low level (Fig 1A), and only two congeners (2,3’,4-trichlorobiphenyl and 2,3,4’-trichlorobiphenyl) were significantly removed when compared to the uninduced control (Table 7). The PCB-degrading pattern of cells grown in a medium containing 0.1 mM of flavanone was very similar to the one obtained for cells grown on sucrose plus 3 mM of biphenyl, as revealed by Tukey’s test (Table 7).

**Table 7:** Effect of flavanone concentration on the PCB-degrading performance of *R*. *erythropolis* U23A.

PCB Congener	% Depletion^a^ of each congener					
	Biphenyl 3mM	Biphenyl 3mM + Sucrose^b^	Sucrose	Flavanone 1 mM + Sucrose	Flavanone 0.1 mM + Sucrose	Flavanone 0.001 mM + Sucrose
3,3'	100±0.0^x^	57±5.2^y^	4.8± 3.1^z^	100±0.0^x^	33.2± 0.5 ^y^	11.7± 6.9 ^z^
4,4'	100±0.0^x^	43.7±1.9 ^y^	4.5± 3.7 ^z^	100±0.0^x^	50.2± 9.7 ^y^	6.1±3.6 ^z^
2,3',4	100±0.0^x^	100±0.0^x^	3.8± 2.2 ^y^	100±0.0^x^	77.0± 1.5 ^z^	85.7±4.3 ^z^
2,4,4'	85± 21^x^	21±8.9 ^y^	9.1± 3.5 ^y^	89.6±9.0^x^	30.5± 4.7 ^y^	5.9±7.0 ^y^
2,3,4'	100±0.0^x^	70±1.6 ^y^	5.5± 3.2 ^z^	93.1± 9.8^x^	54.2± 3.4 ^y w^	46.5±4.6^w^
2,2',3,3'	60.7±15.3^x^	19±3.0 ^y^	0.0± 0.0 ^z^	52.4±12.1^x^	24.8± 4.9 ^y^	0.0±0.0 ^z^
2,3',4,4'	47.6±10^x^	38.7±13.8^x^	0.0± 0.0 ^y^	37.7±7.2^x^	28.5± 1.1^x^	9±4.5 ^y^

^a^ % depletion ± standard deviation, of each congener of a mix of 18 PCB congeners according to the protocol described in the Materials and Methods section. (Only the congeners that are degraded under at least one of the set conditions are listed). Different letters indicate significant differences according to Tukey’s test (P≤0.05)

^b^ Sucrose was added at a concentration of 30 mM in all experiments.

Conversely, the performance of cells grown on sucrose plus 1 mM of flavanone was statistically identical to that of cells grown on 3 mM of biphenyl as a sole growth substrate (Table 7). Therefore, when cells were co-metabolically grown on flavanone plus sucrose, the PCB-degrading performance of strain U23A increased significantly with increasing levels of flavanone, and the data showed that among the tested conditions, optimal PCB-degrading performance was achieved when the cells were grown in the presence of 1 mM of flavanone.

On the basis of the data presented in Fig 1A, we would have expected similar PCB-degrading performances observed for cells grown on 0.1 mM or 1 mM of flavanone. However, unlike the 4-chlorobenzoate production assay, the PCB-degrading assay monitors PCB depletion, which depends solely on the activity of the first enzymes of the pathway. It has been demonstrated that for *P*. *pseudoalcaligenes* KF707 [33], the biphenyl 2,3-dioxygenase and the 2-hydroxy-6-oxo-6-phenyl-hexa-2,4-dienoate hydrolase (the first and fourth enzymes of the pathway) were regulated through different mechanisms. Furthermore, in a previous report [34], benzoate was found to inhibit the 2-hydroxy-6-oxo-6-phenyl-hexa-2,4-dienoate hydrolase. Therefore, we cannot exclude the possibility that the activity and expression of one or more enzymes of strain U23A’s biphenyl pathway may be regulated by a mechanism different from that of the biphenyl dioxygenase.

In spite of this difficulty, our data showed that a concentration of 1 mM of flavanone is required to achieve optimal levels of PCB depletion; at concentrations in the range of 0.001 mM, the pathway was only weakly induced and PCB depletion was much less significant. This explains why in a previous work, using the same PCB-degrading assay [9], we found that when U23A cells were grown in a medium containing 1% of a concentrated *A*. *thaliana* root exudate preparation, only three of the eighteen tested congeners (3,3’-dichlorobiphenyl, 2,3’,4- and 2,3,4’-trichlorobiphenyl) were depleted at a level above control. In that work, we estimated that the concentrated root exudates contained approximately 0.5–1 mM of flavanone, which was the major component [9]. Since the concentrated preparation of exudates was diluted 100-fold in the assay medium, the flavanone concentration was in the order of 0.01–0.005 mM, which was not sufficient to achieve optimal PCB-degrading performance. Therefore, to achieve optimal PCB-degrading (depletion) performance of U23A cells grown directly in the previously described *Arabidopsis* hydroponic culture, plants should have released about 15 times more flavanone than what was observed in that study.

Achieving a variation in this order of magnitude in vegetated soil may not be impossible given the fact that phenylpropanoids released by plants are highly variable and may change substantially according to plant growth conditions [21]. Although we can hardly transpose the results obtained from hydroponic cultures to those in soil, our observations encourage further experimental investigations to determine the various conditions that may influence the ability of PSMs to promote PCB degradation in soil. On the other hand, since many of the flavonoids that might act as inducers of the biphenyl catabolic enzymes are also metabolized by these enzymes, further work will be required to precisely determine how their metabolites may affect the expression and activities of the biphenyl-degrading enzymes.

It is well established that plant exudate composition influences the microbial ecology of the rhizosphere [35]. Unfortunately, the information regarding the amounts and types of chemicals released by plants remains scarce [20, 21, 36]. In the case of flavonoids, their concentrations in the rhizosphere may vary considerably from nanogram to microgram per gram of soil, depending on the plant species, as well as depending on the plant’s age and physiology. Moreover, the concentration of flavonoids also varies along the length of plant roots [21]. Therefore, although some progress has been made with respect to our understanding of how plants may interact with bacteria to promote PCB degradation in soil, the rhizoremediation process will remain empirical until we acquire better insights into the mechanisms that regulate flavonoids and the production of other PSMs, as well as their transport and exudation from plants.

## Conclusion

In the present work we showed that the biphenyl catabolic pathway of the rhizobacterium *R*. *erythropolis* U23A is strongly induced by several flavonoids during co-metabolic growth. However, the PCB-degrading performance of the strain U23A varied significantly depending on the phenylpropanoids concentration in the growth medium. In addition, the ability of phenylpropanoids to induce the biphenyl catabolic pathway was strongly influenced by the co-substrate used as growth substrate. Although many investigations have shown that PSMs may promote the degradation of PCB, the amounts required as well as the plant and bacterial growth conditions required to optimize the effect of PSMs on PCB degradation has not been well studied. Such information is required in order to set up optimal conditions for the rhizoremediation process since in such context, full induction of the biphenyl catabolic pathway will require that the associated plants should release optimal amounts of the appropriate flavonoids as well as the appropriate growth substrates.

In this study we have examined the ability of flavonoids to induce the biphenyl catabolic pathway of a rhodococcal rhizobacterium. Although the role of rhodococcal rhizobacteria in PCB rhizoremediation is not clearly established [11], they were the most abundant bacteria in a PCB-contaminated rhizosphere [37]. On the other hand, in several other studies, the bacteria presumed to play a major role in PCB rhizoremediation were found among the genera *Sphingomonas*, *Burkholderia*, *Pseudomonas*, *Hydrogenophaga Achromobacter*, and *Variovorax* [5, 11, 38–40]. Therefore further insight about how these organisms interact with PSMs is also required to optimize the rhizoremediation process.

Furthermore, the expression and activity of the biphenyl-degrading enzymes may be significantly influenced by as the carbon sources that may act at the transcriptional or posttranscriptional level of the biphenyl catabolic enzymes [22, 23, 41]. It was also found that biphenyl-degrading bacterial strains may respond differently to the various flavonoids and other PSMs with respect to their ability to metabolize them and/or their ability to be induced by them [12, 41, 42]. In addition, the success of rhizoremediation processes may vary according to the plant species growing on contaminated sites [3, 43]. Therefore, our work emphasizes the need for acquiring better insights about the various factors influencing PSMs released by plants and about how all known rhizobacteria interact with PSMs and how they compared to each other’s.
